# Editorial: Soil microbes along elevational gradients: community structure, diversity, and role in nutrient cycling

**DOI:** 10.3389/fmicb.2023.1266325

**Published:** 2023-08-10

**Authors:** José A. Siles, María Gómez-Brandón, Heribert Insam, Maraike Probst

**Affiliations:** ^1^Department of Soil and Water Conservation and Organic Waste Management, Center for Edaphology and Applied Biology of Segura - Spanish National Research Council (CEBAS-CSIC), Murcia, Spain; ^2^Grupo de Ecoloxía Animal, Universidad de Vigo, Vigo, Spain; ^3^Institut für Mikrobiologie, Universität Innsbruck, Innsbruck, Austria

**Keywords:** microbial community, microbiome, altitudinal gradient, environmental gradient, modeling

Microbes play key roles in soil. It is known that climatic factors, edaphic properties, and plant communities influence soil microbial diversity and community composition (Delgado-Baquerizo et al., 2016; Köninger et al., 2022). Nonetheless, if we aim at incorporating soil microbial features into ecosystem models to improve their predictive capacity, a deeper understanding of the relationships between soil microorganisms, vegetation, and soil properties is needed (Fry et al., 2019). In this context, elevational gradients have been recognized as useful “natural experiments” to assess the effect of a wide range of environmental factors on soil microbial communities since they are characterized by dramatic changes in climate and biotic characteristics over short geographic distances (Körner, 2007).

In the last few years, there has been an important surge in studies on soil microbes and elevation. Different patterns of soil microbial diversity and abundance with elevation have been identified, which are driven by temperature, precipitation, soil pH, nutrient contents, carbon/nitrogen ratio, and plant productivity, depending on the given gradient and its geographical location; however, absence of such patterns has also been reported (Looby and Martin, 2020). This points out the need of further studies. Furthermore, soil nutrient contents and chemical composition of soil organic matter change with elevation (Bardelli et al., 2017; Siles et al., 2017). Understanding how these changes are controlled by soil microbes, and *vice versa*, is relevant for state-of-the-art ecological models. In this context, the present Research Topic was motivated.

The objective of this Research Topic was to provide a platform to researchers for sharing their new studies on soil microbes along elevational gradients and their driving factors. This Research Topic was especially interested in compiling novel information on seasonal dynamics, network structure, and functionality of soil microbial communities and litter decomposition along elevational gradients across the planet. The present Research Topic comprises eight original research articles, contributed by 57 authors.

Three articles were focused on characterizing soil bacterial and/or fungal communities and their driving factors along different elevational gradients. Díaz et al. investigated soil bacterial communities (based on metabarcoding) along an elevational gradient [4,783 to 5,583 m above sea level (asl)] in an Andean glacier in Ecuador. The authors found a trend toward higher diversity at lower elevations for glacier ice and soil samples and changes in bacterial community structure were not strongly explained by soil nutrient contents. The work by Feng et al. explored soil bacterial and fungal communities (metabarcoding) along a tropical elevational gradient (265–1,400 m asl) located in Hainan Island, China. The authors found that bacterial and fungal diversity declined as elevation increased. Soil pH was found to be the major driver of bacterial diversity, while soil moisture was the best predictor of soil fungal diversity. The authors suggested that local-climate variability plays a crucial role in structuring the distribution of soil microbial communities along the studied tropical montane gradient, which supports Janzen's mountain passes hypothesis. Liu et al. studied abundance (phospholipid fatty acid content) and potential enzyme activities of soil microbial communities along an elevational gradient (2,820–4,280 m asl) in eastern Qinghai-Tibetan Plateau. While microbial biomass was higher at the lowest elevation, the enzyme activities exhibited the maximum values at the highest elevation. These contrasting responses were explained by the different adaption strategies of soil microbes to population growth and resource acquisition.

Two articles were more focused on studying the responses of soil microbial communities to elevation from a functional perspective. Zhao M. et al. investigated the concentrations and metabolic characteristics of type I (16:1ω7c and 16:1ω5c) and type II (18:1ω7c) methanotrophs in peatlands along an elevational gradient (300–1,500 m asl) in the Changbai Mountain, China. They found that while type I methanotrophs dominated at lower elevations, type II methanotrophs dominated at higher elevations. Analysis of δ^13^C isotopes showed that the two microbial groups had different carbon preferences. Merges et al. combined comparative and population genomics to assess the presence and absence of genes in high and low elevation genomes of the lichenized fungi *Umbilicaria phaea* and *Umbilicaria pustulata*. The authors found 16 orthogroups with shared orthologous genes in genomes at low elevation and 13 at high elevation. This work provides insights into gene content variation of lichenized fungi in relation to elevation-related climatic gradients.

Zhao Y. et al. collected 199 samples from an elevational gradient (1,500–2,400 m asl) in the Huoditang forest in the Qinling Mountains, China. Based on 16S rDNA, kriging interpolation, geostatistics, and soil properties, they observed that the most abundant bacterial phyla were highly correlated to spatial distances. Soil nutrients (36.5%), especially soil organic carbon and available phosphorous, environmental factors (28.2%), mainly pH, and topographic factors (7.8%), especially elevation, explained the variation in bacterial community structure.

The decomposition of standardized leaf organic matter was studied by Semeraro et al. as a function of 19 biotic and abiotic variables. Across 24 sites, these authors exposed leaf litter bags (teabags) to two distinct bioclimatic regions (Swiss northern Prealps and southern Alps), two elevations (1,500 and 2,100 m asl), and two expositions (north and south slope). While several variables were relevant predictors for the decomposition of organic matter decomposition, the primary predictors were the pedo-climatic niche and solar radiation. The study from Xu et al. focused on the biodiversity patterns of rhizosphere microorganisms and their host plants along elevational gradients in tropical, subtropical, and subalpine forests in Yunnan Province, China. These authors observed that environmental factors like elevation, temperature, or humidity had a stronger direct effect on rhizosphere microbial diversity than on host plant diversity. Moreover, they reported divergent elevational patterns and environmental responses of soil microorganisms depending on their functional group (e.g., pathogen, mycorrhiza and nitrifier).

The articles published in this Research Topic represent a step forward in the understanding of the taxonomic and functional responses of soil microbes to the environmental factors changing along elevational gradients. The knowledge provided here will be useful to conduct global meta-analyses on soil microbes and elevation and to develop more accurate ecosystem models.

## Author contributions

JS: Writing—original draft, Writing—review and editing. MG-B: Writing—original draft, Writing—review and editing. HI: Writing—original draft, Writing—review and editing. MP: Writing—original draft, Writing—review and editing.
